# Supplementary material for: Sensorimotor pathway controlling stopping behavior during chemotaxis in the Drosophila melanogaster larva
Source: eLife. 2018 Nov 22;7:e38740. doi: 10.7554/eLife.38740 (PMC6264072; doi:10.7554/eLife.38740)
Supplement: Transparent reporting form [file elife-38740-transrepform.pdf]

eLIFE
1st Floor
24 Hills Road
Cambridge CB2 1JP, UK
P 01223 855340
W elifesciences.org
T @elife

eLife's transparent reporting form

We encourage authors to provide detailed information *within their submission* to facilitate the interpretation and replication of experiments. Authors can upload supporting documentation to indicate the use of appropriate reporting guidelines for health-related research (see EQUATOR Network), life science research (see the BioSharing Information Resource), or the ARRIVE guidelines for reporting work involving animal research. Where applicable, authors should refer to any relevant reporting standards documents in this form.

If you have any questions, please consult our Journal Policies and/or contact us: editorial@elifesciences.org.

Sample-size estimation

- You should state whether an appropriate sample size was computed when the study was being designed
- You should state the statistical method of sample size computation and any required assumptions
- If no explicit power analysis was used, you should describe how you decided what sample (replicate) size (number) to use

Please outline where this information can be found within the submission (e.g., sections or figure legends), or explain why this information doesn't apply to your submission:

In this work, we chose sample sizes that were sufficiently large to ensure both the significance and the power of comparisons between the means of different samples (i.e., minimization of type I and type II errors). For samples distributed according to normal distributions, the "sampsizepwr" function from the Statistics toolbox in Matlab (Mathworks, MA) was used for sample-size estimations. To estimate the sample size (n_0), we combined a standard power level of 0.8 with a suspected mean (μ) and standard deviation (σ), and desired difference (δ). Suspected means, standard deviations and δ differences were derived from pilot behavioral experiments. For loss-of-function experiments, statistical power of 0.8 could be achieved for turn rate comparisons by using a sample size of 18 larvae. For gain-of-function experiments, n_0=33 trials led to the statistical power of 0.8 in statistical comparisons of stopping probabilities. To err on the conservative side, the final sample size for each experiment was chosen to be larger than the sample-size estimation.

For samples distributed normally, the power of each statistical test was calculated with the sample size, mean, and standard deviation of the control group, and the difference between the control and test groups. For the experiments with more than one pairwise comparison, the power analysis was based on the least favorable pairwise comparison. There was only one sample group that was distributed normally (Figure 1 — Figure Supplement 3). n_0=36 fulfilled the statistical power of 0.8 for these experiments. We ensured that sample sizes were sufficiently large so that the power of each test exceeded 0.8. Power analysis could not be achieved for comparisons involving samples that departed from normal distributions. Due to the complexity of functional-imaging manipulations and the analysis of wave propagation in restricted larvae, we had to use smaller sample sizes for these experiments. As explained in the Materials and Methods section, normality of the samples was assessed by the Lilliefors test at $p<0.01$.

eLife Sciences Publications, Ltd is a limited liability non-profit non-stock corporation incorporated in the State of Delaware, USA, with company number 5030732, and is registered in the UK with company number FC030576 and branch number BR015634 at the address 1st Floor, 24 Hills Road, Cambridge CB2 1JP | August 2014

Replicates

- You should report how often each experiment was performed
- You should include a definition of biological versus technical replication
- The data obtained should be provided and sufficient information should be provided to indicate the number of independent biological and/or technical replicates
- If you encountered any outliers, you should describe how these were handled
- Criteria for exclusion/inclusion of data should be clearly stated
- High-throughput sequence data should be uploaded before submission, with a private link for reviewers provided (these are available from both GEO and ArrayExpress)

Please outline where this information can be found within the submission (e.g., sections or figure legends), or explain why this information doesn't apply to your submission:

The size of every data sample included in the study is reported in the Figure legends. For loss-of-function experiments, we could not perform technical replicates since each larva could be tested only once. For gain-of-function and imaging experiments, technical replicates were performed and the number of replicates were indicated in the Figure legends. All trials involved animals that behaved independently of each other except for the loss-of-function screen where groups of ~20 larvae were tested in the same assay. For the loss-of-function screen, which involved tracking of multiple larvae, we picked independent trajectories for further analysis. As described in the manuscript, our analysis pertains to means or medians computed over the behavior of different larvae. All data points and their identity (e.g. independent larvae, trial, trajectories etc.) were shown in the box plots and indicated in the Figure legends. Outliers reported in boxplots were defined as follows: data points were considered outliers if they are greater than $q_3 + w \times (q_3 - q_1)$ or less than $q_1 - w \times (q_3 - q_1)$, where w is the maximum whisker length, and q_1 and q_3 are the 25th and 75th percentiles of the sample data, respectively. The whisker length w was determined by the default settings of the "boxplot" function of Matlab_R2015b. Following the behavioral experiments, individual larvae were inspected based on the analysis of the trajectories and kinematic variables. Larvae that showed long periods of inactivity or larvae that left the behavioral arena prematurely (<30s after the beginning of the trial) were excluded from the analysis. These cases very less than 5-10% of the whole dataset. Long periods of inactivity were thought to be due to accidently harsh mechanical manipulation of the larvae during their introduction in the arena. Larvae tended to leave the behavioral arena prematurely if they were not oriented towards the odor source upon their introduction in the arena. Occasionally (<5% of trials), tracking was hampered because of irregularities in the agarose, distortion of the image (due to the presence of odor source in Figures 4J-M) or due to poor contrast. These trials were also excluded from the dataset. For the functional imaging experiments, the CNS preparations that did not show any motor neuron activity prior to optogenetic stimulation were excluded from the dataset. Lack of activity in these preparations was thought to be due to improper microdissection of the CNS.

Statistical reporting

- Statistical analysis methods should be described and justified
- Raw data should be presented in figures whenever informative to do so (typically when N per group is less than 10)
- For each experiment, you should identify the statistical tests used, exact values of N, definitions of center, methods of multiple test correction, and dispersion and precision measures (e.g., mean, median, SD, SEM, confidence intervals; and, for the major substantive results, a measure of effect size (e.g., Pearson's r, Cohen's d)
- Report exact p-values wherever possible alongside the summary statistics and 95% confidence intervals. These should be reported for all key questions and not only when the p-value is less than 0.05.

Please outline where this information can be found within the submission (e.g., sections or figure legends), or explain why this information doesn't apply to your submission:

The statistical analysis was described in the "Statistical procedures" section of the Materials and Methods. Samples that could not be modeled as normal distributions were represented by box plots. The statistical tests, multiple test corrections and sample sizes used throughout the study were reported in the Figure legends. All p-values can be found in Supplementary File 1.

(For large datasets, or papers with a very large number of statistical tests, you may upload a single table file with tests, Ns, etc., with reference to sections in the manuscript.)

Additional data files ("source data")

- We encourage you to upload relevant additional data files, such as numerical data that are represented as a graph in a figure, or as a summary table
- Where provided, these should be in the most useful format, and they can be uploaded as "Source data" files linked to a main figure or table
- Include model definition files including the full list of parameters used
- Include code used for data analysis (e.g., R, MatLab)
- Avoid stating that data files are "available upon request"

Please indicate the figures or tables for which source data files have been provided:

Our behavioral experiments rely on the quantification of the time series of larval postures segmented online during tracking with a customized closed-loop tracker described in (Schulze et al, eLife 2015). Functional imaging data rely on the analysis of image series obtained by confocal/two-photon scanning microscopy. Analysis of the gain-of-function in restricted larvae relies on raw movie sequences. We performed in-depth analysis of the un-processed time series, which will be made available on the GitHub account of the Louis lab (wherever feasible). As we have done with our previous manuscripts (including those previously published in eLIFE), we will make annotated versions of the main Matlab data-analysis scripts accessible on our lab's Github account upon acceptance of the manuscript.
